# Concerns about stone free rate and procedure events of percutaneous nephrolithotripsy (PCNL) for 2–4 cm kidney stones by standard-PCNL vs mini-PCNL- comparative randomised study

**DOI:** 10.1186/s12894-023-01270-1

**Published:** 2023-05-19

**Authors:** Mohamed Wishahi, Ahmed El Feel, Amr Elkhouly, Abdullah Fahmy, Mamdouh Roshdy, Ahmed G Elbaz, Ahmed I Kamel, Mohamed Badawy, Khaled Elesaily, Samir Eldahshan, Ayman Ali, Ahmed Meheina, Mohamed Abdelwahed

**Affiliations:** 1 Department of Urology, Theodor Bilharz Research Institute, Cairo, Egypt; 2 Department of Urology, Faculty of Medicine, Kasr Alaini Medical School, Cairo University, Cairo, Egypt

**Keywords:** Percutaneous nephrolithotomy, Renal stones, Mini percutaneous nephrolithotomy, Stone free rate, Kidney stones

## Abstract

**Background:**

To compare the efficacy and safety of standard percutaneous nephrolithotomy (PCNL) with mini- PCNL for kidney stones 2–4 cm.

**Methods:**

Eighty patients were enrolled in a comparative study, they were randomly divided into mini-PCNL group (n = 40) and standard-PCNL (n = 40). Demographic characteristics, perioperative events, complications, stone free rate (SFR) were reported.

**Results:**

Both groups showed no significant difference in clinical data about age, stone location, back pressure changes, and body mass index. The mean operative time was (95 ± 17.9 min) in mini-PCNL, and (72.1 ± 14.9 min). Stone free rate were 80% and 85% in mini-PCNL and standard-PCNL respectively. Intra-operative complications, post-operative need for analgesia, hospital stay were significantly higher in standard-PCNL compared to mini-PCNL (85% vs. 80%). The study followed CONSORT 2010 guidelines for reporting parallel group randomization.

**Conclusion:**

Mini-PCNL is an effective and safe treatment of kidney stones 2–4 cm, it has the advantage over standard-PCNL being has less intra-operative events, less post-operative analgesia, shorter hospital stay, while operative time and stone free rate are comparable when considering multiplicity, hardness, and site of stones.

## Background

Since the introduction of percutaneous nephrolithotripsy (PCNL) in 1980 the, the procedure gained popularity and steady development in nephroscope, energy source for stone fragmentation, patient position, puncture site, tract dilatation, smaller nephrostomy tube or tubeless PCNL. The Second wave of development was the miniaturization of instruments that shifted the access sheath of 30 French (Fr) in standard-PCNL, to 14–20 Fr in mini-PCNL [1].

Mini-PCNL in adults showed good stone free rates (SFR), minimal risk of bleeding, decreased hospital stay and reduced the need for postoperative analgesia [2].

Hu G et al reported in a series of 1368 patients undergoing mini-PCNL using 16 Fr tract and stone disintegration within holmium laser or pneumatic lithoclast, the SFR was 82% [3].

Comparing bleeding complications in mini-PCNL and Standard-PCNL, it was lower in mini-PCNL (1.4%) [4].

Mini-PCNL and retrograde intrarenal surgery (RIRS) became the priority in the treatment of kidney stones, both are safe and effective methods particularly for treating lower pole stones with a diameter of 1.5–2.5 cm [5].

Standard-PCNL is considered in recent years an old procedure and would be replaced by mini-PCNL and RIRS, but it has its role in the treatment of large and complex kidney [6].

Considering SFR, it was significantly higher in standard-PCNL compared to mini-PCNL in adult patients with stones less than 2cm [7]. Patients having kidney stones more than 2 cm, it is recommended to compare the safety and efficacy of these two procedures [8].

PCNL has a minimal impact on global kidney function in the location of the procedure. Kidneys with some degree of impaired function would benefit from PCNL to alleviate obstruction and protected against stone complications [9].

Zeng et al made a comparison of mini-PCNL and Standard-PCNL for kidney of stones > 2cm, they analyzed the data regarding SFR, intra operative complications, operation time, postoperative outcome, and hospital stay, they concluded that mini-PCNL is an effective treatment of > 2cm kidney stones and is equal to standard-PCNL, both have a comparable SFR, with relative advantage of mini-PCNL of less blood loss and shorter hospitalization [10].

## Patients and methods

A randomized prospective comparative study was conducted to enroll 80 patients with kidney stones 2–4 cm in its longest diameter whether solitary or multiple during the period from August 2021 till November 2022. All procedures performed in studies involving human participants were in accordance with the ethical standards of the institutional research committee and with the 1964 Helsinki Declaration and its later amendments. The study protocol was approved by Medical Research Ethics Committee, Faculty of Medicine, Cairo University (MS-571-2021). Informed consent was signed by all patients after explaining the benefits and risks of each procedure.

*Randomization and allocation* Randomization started with flip coin method in the initial randomization and continued with an alternative method, randomization was done by an author (MB) who was blinded of the clinical characteristics to distribute the patients to either mini-PCNL or standard-PCNL, this. Mini-PCNL group included 40 patients (28 males and 12 females). Standard-PCNL group included 40 patients (20 males and 20 females).

*Preoperative protocol* Preoperative evaluation included physical examination, body mass index (BMI), skeletal deformities and previous kidney intervention. Preoperative laboratory studies included urine culture and antibiotic sensitivity, complete blood count (CBC), serum creatinine, blood urea nitrogen, serum uric acid, blood sugar, and coagulation profile. Imaging included plain X-ray to abdomen (KUB), and non-contrast spiral computerized tomography (CT urography). Stone size was assessed by measuring the longest diameter of the largest stone.

*Inclusion and exclusion* Inclusion criteria were adult patients undergoing PCNL for renal stones 2–4 cm. Exclusion criteria were urinary tract infection, age below 18 years, uncorrected coagulopathy.

*Surgical procedure* The procedures of mini-PCNL and standard-PCNL were performed by endoulology surgical team formed of 5 expertise who had vast experience and similar learning curve.Patients underwent the procedure under general anesthesia; cystoscopy was performed to advance a 6 Fr open-ended ureteral catheter under fluoroscopic guidance to the collecting renal system. All patients were placed in supine position with 30 degrees tilting. Renal access was achieved under fluoroscopy after opacification of the collecting system by injection of diluted contrast medium via the ureteral catheter. For localization puncture we used triangulation guided with fluoroscopy and pre-operative CT images in the coronal, axial, and the reconstruction images. The puncture site was the lower posterior calyx in both mini-PCNL and standard-PCNL, 5 cases needed second puncture, 2 cases of mini-PCNL, and 3 cases of standard-PCNL, those who needed second puncture, it was in the middle calyx. The puncture is followed with insertion of curved floppy tip guide wire (0.038 inch) that will guide the track dilatation. Tract dilatation for mini-PCNL was up to 14–16 Fr, and 30 Fr for standard-PCNL, tract dilatation was done to by Teflon facial dilators (Cook, Inc.) with acute single step. Twelve Fr rigid nephroscope (Karl Storz, Tuttlington, Germany) was used for mini-PCNL, for standard PCNL, 28 Fr rigid nephroscope (Karl Storz, Tuttlington, Germany) was used. In both mini-PCNL and standard-PCNL stone disintegration was achieved with combination of Holmium Laser and pneumatic lithotripter, at the end of the procedure nephrostomy tubes were placed through the nephrostomy tract. Tubeless PCNL was not applies in our cases.

In the first post-operative day an abdominal plain X-ray and laboratory work-up are done. Postoperative pain was assessed according to the visual analogue scale and the need of analgesic. Patients received antibiotics for 5 days postoperatively.

Perioperative complications were assessed according to the modified Clavien grading system. Follow-up was done after 3 weeks with CT urography to evaluate SFR, residual fragments smaller than 3 mm were considered stone-free.

*Statistical analysis* Statistical analysis was conducted using SPSS 22nd edition, quantitative data were presented in mean and standard deviation for parametric data and in median and range for non-parametric data after normality testing. Comparison of quantitative variables between study groups was conducted using student T test for parametric variables and Mann Whitney U test. Qualitative variables were presented in frequency and percentages, p value < 0.05 was considered statistically significant.

## Results

Eighty patients were prospectively randomized into two groups to undergo mini-PCNL (n = 40) or standard-PCNL (n = 40). Mean age 41.6 ± 12.1 years, males were 60% while females were 40%.

Comparison of age, gender, BMI, and chronic illnesses showed no statistically significant difference between the two study groups with p-values > 0.05.There was no statistically significant difference between the treatment groups in preoperative values, and X-ray exposure time with p-values > 0.05. (Table 1). No significant difference was found between study groups in terms of stone characteristics including stones size, multiplicity, and location (Table 2).

**Table 1:** Demographics data

		Groups				P value
		mini-PCNL n = 40		Standard PCNL n = 40		
		Mean/ Count	SD/ %	Mean/ Count	SD/ %	
Age (years)		39.5	11.5	43.7	12.7	0.280
BMI (kg/m2)		28.2	3.6	27.3	4.7	0.583
Sex	Female	12	30.0%	20	50.0%	0.197
	Male	28	70.0%	20	50.0%	
Medical history	Free	36	90.0%	30	75.0%	0.138
	CKD	0	0.0%	0	00.0%	
	DM	0	0.0%	6	15.0%	
	HTN	2	5.0%	4	10.0%	
	IHD	2	5.0%	0	0.0%	

CKD, chronic kidney disease; DM, diabetes mellitus; HTN, hypertension

IHD, ischemic heart disease

**Table 2:** Stone characteristics and operative details of PCNL

Variables	Mini-PCNL	Standard-PCNL
Number of cases	n = 40	n = 40
Stone size	2–4 cm	2–4 cm
Single Stone/ multi-calyceal Single stone Big stone& Multiple calyceal Patient positioning Localization method: Triangulation guided with fluoroscopy and pre-op CT	22 18 Supine 40	19 21 Supine 40
Puncture site Single puncture: lower posterior calyx Second puncture: middle calyx	38 2	37 3
Nephroscope size	12	28
Stone disintegration equipment: Holmium Laser & Pneumatic	40	40
Perforation & management	1 Double J insertion	3 Double J insertion
Nephrostomy tube insertion	40	40
Intra-operative bleeding	0	1
Operation time -minutes(Mean)	95.0	72.1

Operative time was less among standard-PCNL compared to mini-PCNL (72.1 ± 14.9 minutes versus 95 ± 17.9 minutes) with p-value 0.0001. This difference can be explained with the variety of cases in regard to size, multiplicity and the need to have a second puncture.

Intraoperative Complications: Among mini-PCNL group, there was one patient that had collecting system perforation that was managed with insertion of double J stent, while patients’ undergone standard-PCNL, three of them had collecting system perforation, that was managed with insertion of double j stent. In the 4 patients the perforations regress spontaneously and leakage stopped in the second post-operative day.

One patient among standard-PCNL had intraoperative bleeding, that showed decreased hemoglobin level (Hb) and hematocrit value, the anesthetist recommended and gave the blood transfusion.

On the other hand, there was no intraoperative bleeding in mini-PCNL patients. Blood loss in terms of the (mean ± SD) change in Hb level on first postoperative day in mini-PCNL was (0.58 ± 0.64) gm/dl which was significantly lower (P < 0.05) when compared to standard-PCNL (1.64 ± 0.93) gm/dl.

Postoperative pain was higher in standard-PCNL (7.5 ± 0.7) versus (5.7 ± 1.1) in mini-PCNL with p values 0.0001.

The need for non-steroidal anti-inflammatory drugs was higher in standard-PCNL compared to mini-PCNL.

**Fig. 1:**
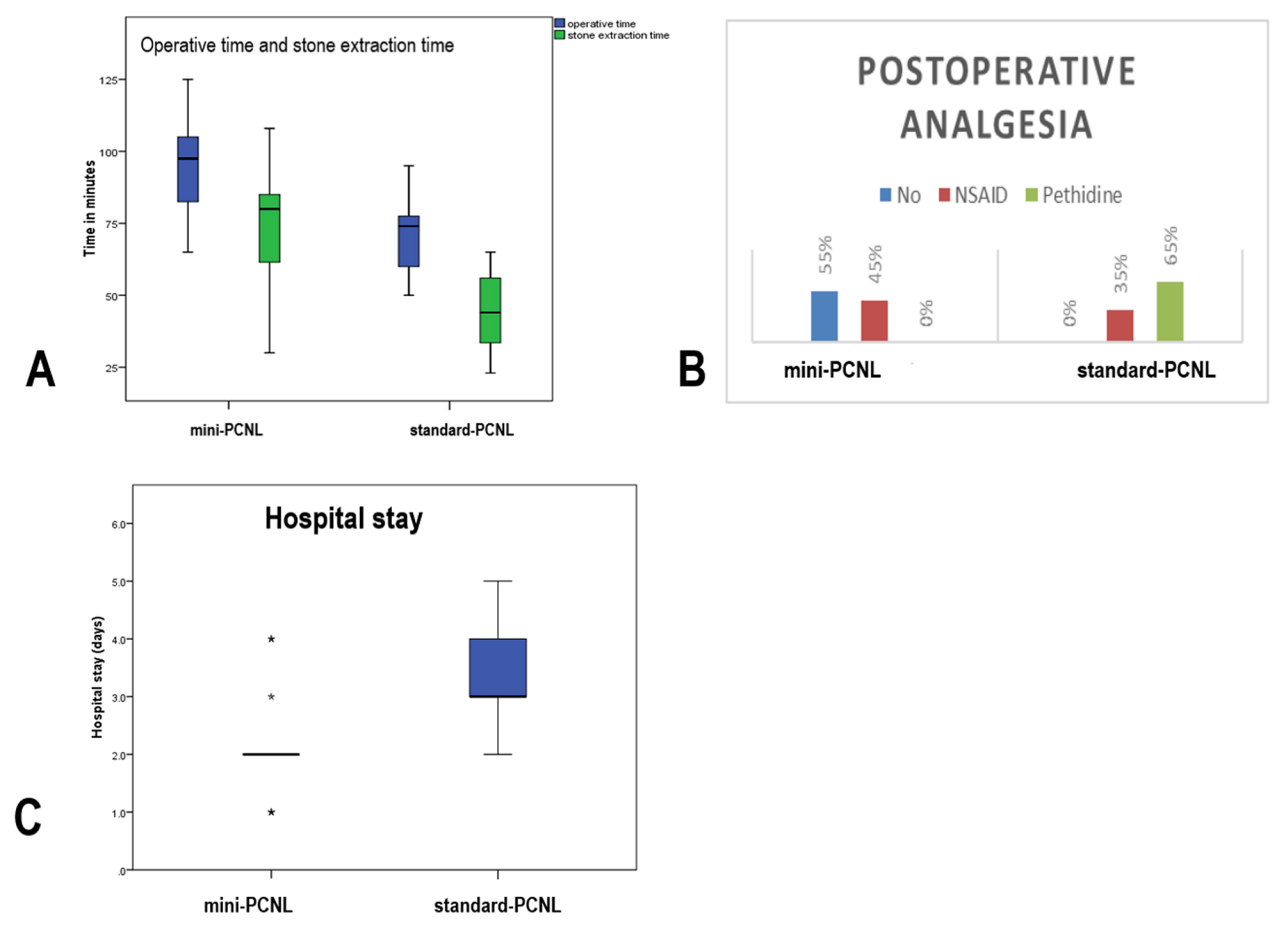
Operative and postoperative outcome in mini-PCNL vs standard-PCNL: (**A**) Showing difference in Operative time and stone extraction time between mini-PCNL vs mini-PCNL. (**B**) Showing the need for postoperative analgesics among mini-PCNL vs standard-PCNL. (**C**) Showing hospital stay (days) among mini-PCNL vs standard-PCNL

Hospital stay was longer in standard-PCNL compared to mini-PCNL (3.4 +/- 1.1 days versus 2.05 +/- 0.9 days) with p-value 0.0001. Stone free rate in mini-PCNL group was 80% versus 85% in standard-PCNL however; this difference was not statistically significant (Table 3, Fig. 1).

**Table 3:** Preoperative, intraoperative, and postoperative parameters

			Mini-PCNL (n = 40)		standard-PCNL (n = 40		P value
			Mean/ Count	SD/ %	Mean/ Count	SD/%	
**Preoperative parameters** Hb (gm/dl)			13.2	1.3	13.3	1.5	0.877
Serum Creatinine (mg/dl)			1.09	.28	1.26	.58	0.251
Blood urea (mg/dl)			28.6	7.2	34.5	17.7	0.172
**Operative time (minutes)**			95.0	17.6	72.1	14.9	0.0001
**X-ray exposure time (seconds)**			344.0	59.5	314.0	84.2	0.201
**Intra-operative complications**		None	39	95.0%	36	90.0%	0.626
		Collecting system perforation (DJ fixation)	1	2.5%	3	7.5%	
		intraoperative bleeding	0	0.0%	1	2.5%	
**Post-operative parameters** Hb drop (gm/dl) Change in Creatinine(mg/dl)			0.58 0.0	0.64 0.11	1.64 0.07	0.93 0.24	0.0001 0.272
**Postoperative pain** **(Visual analogue scale of pain)**			5.7	0.7	7.5	1.1	0.0001
**Postoperative** **Need for analgesia**	No		22	55.0%	0	0.0%	0.0001
	NSAID		18	45.0%	14	35.0%	
	Pethidine		0	0.0%	26	65.0%	
**Hospital stays (days)**			2.05	0.9	3.4	1.1	0.0001
**Stone free rate**			32	80.0%	34	85.0%	0.677

## Discussion

The present study is a prospective comparative study that was conducted on 80 patients who had radio-opaque renal stones 2–4 cm, they were randomized to undergo either mini-PCNL or standard-PCNL.

Characteristics of the patients included in the study showed no significant difference regarding mean age, gender, mean BMI and mean stone size. The endpoints of the study were: operative time, blood loss, Intraoperative and postoperative events, stone free rate, hospital stay, and postoperative pain. Operative time in mini-PCNL and standard-PCNL was calculated from the time of cystoscopy till securing the nephrostomy tube.

The mean operative time for mini-PCNL was (95 ± 17 minutes) which was longer than the operative time in standard-PCNL (72 ± 14.9 minutes), these results are similar to study by Qin et al [10]. Zhu et al compared mini- PCNL vs standard-PCNL. It was concluded that operative time was shorter in the standard-PCNL group [8]. In our series, and in other studies, the operative time cannot be considered as an absolute criteria of advantage of one technique over the other, although there are criteria in selection as stone size, but other criteria are variable from one case to the other, these element includes multiplicity, harness of the stone, location, and calyceal stone.

Hospital stays calculated from the day of surgery to the day of discharge was (2.05 ± 0.9) days for mini-PCNL patients, compared to (3.4 ± 1.1) days in standard-PCNL. Zhu et al in a Systematic review and meta-analysis showed a shorter hospitalization for mini- PCNL group [8]. The present study showed that hospital stay was shorter in mini-PCNL group compared to standard-PCNL, this finding imply a great advantage of the mini-PCNL for the patients and in reducing costs of the procedure.

Blood loss was assessed by comparing the pre and post-operative hemoglobin level in day one postoperative. Blood loss in terms of the mean change in Hb level in mini-PCNL group was (0.58 ± 0.64) gm/dl which was lower when compared to standard-PCNL group (1.64 ± 0.93) gm/dl. Mishra et al compared mini-PCNL and standard-PCNL for renal stones (1–2 cm), shows that mini PCNL has the advantage of reduced hemoglobin drop (0.8 ± 0.9 vs.1.3 ± 0.4) [2], their data is confirmed with our results that blood loss was less in mini-PCNL in comparison to standard-PCNL.

In present study there was statistically significant difference between the two groups regarding total complication rate, where the intra-operative bleeding was reported in standard-PCNL, perforation of the urinary system was reported 3 timed in standard-PCNL compared to one case in mini-PCNL(Table 2), our results are comparable with the results of Zhu et al and Qin et al [8, 10].

In the present study there were no changes of kidney function in mini-PCNL or standard-PCNL compared to pre-operative parameters. Earlier reports showed that PCNL has a minimal impact on global kidney function, could be conducted even in kidneys with some degree of functional impairment, and the procedure is recommended to prevent the development of complications.

Postoperative pain was higher among standard-PCNL (7.5 ± 1.1) versus (5.7 ± 0.7) in mini-PCNL with p values 0.0001. The systematic review and meta-analysis of Zhu et al [8] showed that only 3 studies were assessing postoperative pain using VAS and it was statistically significantly lower among mini- PCNL group. Same results were confirmed by Zeng et al [11], their data is in accordance of our finding that post-operative pain in mini-PCNL was lower compared to standard-PCNL.

The SFR was 80% and 85% in mini-PCNL and standard-PCNL respectively, this difference was not statistically significant. Zeng et al in a multicenter study comparing mini-PCNL versus standard-PCNL in treatment of renal stones larger than 2 cm, stated that SFR of mini-PCNL and standard-PCNL were presumed to be 83% and 89% [21]. Zhu et al reported that there was no difference between mini PCNL and standard PCNL in terms of stone free rate [8].The present study showed that SFR was comparable in both groups.

Definition of the SFR in different studies was slightly variable; in the present study a residual fragment less than 3mm with no stone related events were defined as SFR.

In the present study patients who undergone mini-PCNL or standard-PCNL did not encounter residual stones related events.

## Limitation

The limitation of the present study is that it did not have longer follow-up to evaluate the fate of residual fragments, and the number of cases is not comparable to that of other big series.

## Conclusion

Mini-PNL is significantly more advantageous in terms of hemoglobin drop, length of hospital stay, need for analgesics, and postoperative pain. Regarding stone free rate and operation time they are close to each other with small difference in both procedure, these difference can be attributed to non-standarsation of cases in regard to multiplicity of stones, stone hardness, stone location, and need for a second puncture. Standard-PCNL had a higher complication rate compared to mini-PCNL. Our study showed that a successful safe mini-PCNL required an accurate evaluation of the case with consideration of second puncture for inaccessible calcyeal stones, the use of pneumatic lithotripter for initial fragmentation of the big stones to be followed by holmium laser for dusting of the stone fragments, cases that showed perforation of the system would managed successfully with insertion of double J stent.

## Data Availability

All data generated or analyzed during this study are included in this published article Competing interests

## Abbreviations

PCNL: percutaneous nephrolithotripsy
FR: French scale
SFR: stone free rates
CBC: complete blood count
CT urography: spiral computerized tomography
TLA: Trans lumbar angiography needle
